# (–)-Xanthatin as a Killer of Human Breast Cancer MCF-7 Mammosphere Cells: A Comparative Study with Salinomycin

**DOI:** 10.3390/cimb44090264

**Published:** 2022-08-25

**Authors:** Shuso Takeda, Masayo Hirao-Suzuki, Mitsuru Shindo, Hironori Aramaki

**Affiliations:** 1 Laboratory of Molecular Life Sciences, Faculty of Pharmacy and Pharmaceutical Sciences, Fukuyama University, Sanzou 1, Hiroshima 729-0292, Japan; 2 Laboratory of Xenobiotic Metabolism and Environmental Toxicology, Faculty of Pharmaceutical Sciences, Hiroshima International University, 5-1-1 Hiro-koshingai, Hiroshima 737-0112, Japan; 3 Institute for Materials Chemistry and Engineering, Kyushu University, 6-1 Kasuga-koen, Kasuga 816-8580, Japan; 4 Department of Molecular Biology, Daiichi University of Pharmacy, 22-1 Tamagawa-cho, Fukuoka 815-8511, Japan

**Keywords:** (–)-xanthatin, cancer stem cells, mammospheres, MCF-7 cells, GADD45G

## Abstract

Experimental evidence accumulated by our research group and others strongly suggests that (–)-xanthatin, a xanthanolide sesquiterpene lactone, exhibits anti-proliferative effects on human breast cancer cells (in vitro) as well as anti-tumor effects in experimental animals (in vivo). In cancer biology, it is now critically important for anti-cancer agents to selectively target cancer stem cells (CSCs) in order to overcome cancer therapeutic resistance and recurrence. However, it has not yet been established whether (–)-xanthatin abrogates the formation of breast CSCs. In the present study, we utilized chemically synthesized pure (–)-xanthatin and a culture system to obtain mammospheres from human breast cancer MCF-7 cells, which are a CSC-enriched population. We herein demonstrated for the first time that (–)-xanthatin exhibited the ability to kill mammospheres, similar to salinomycin, an established selective killer of CSCs. A possible anti-proliferative mechanism toward mammospheres by (–)-xanthatin is discussed.

## 1. Introduction

**Figure 1:**
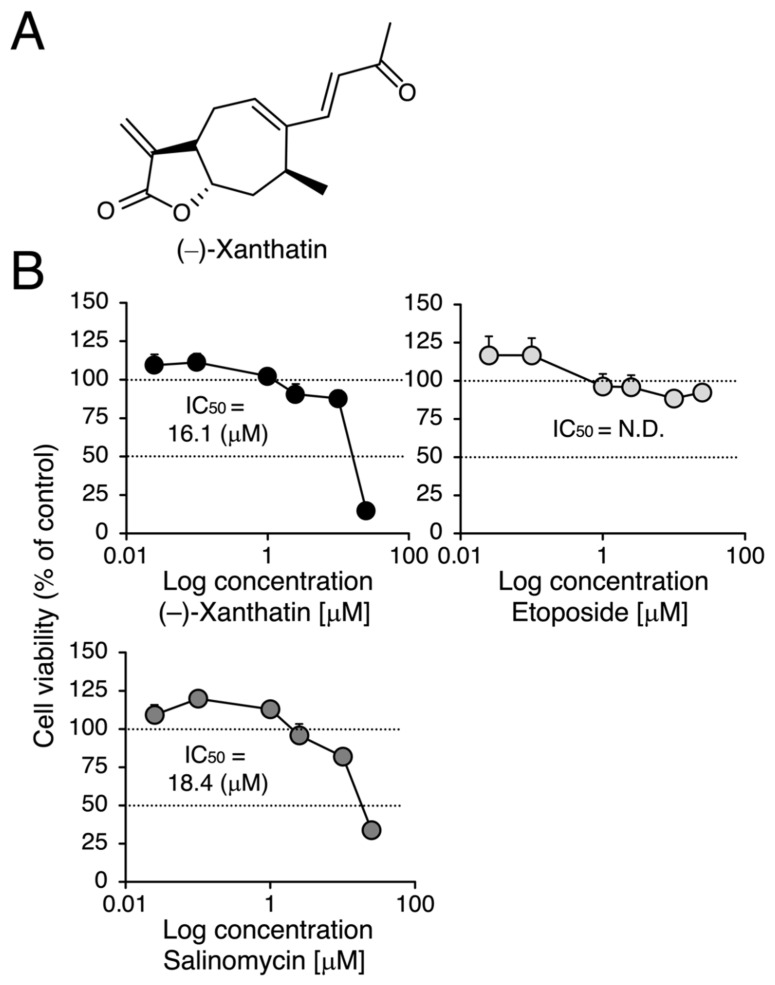
Effects of (–)-xanthatin, etoposide, and salinomycin on the viability of MCF-7 cells. (**A**) The chemical structure of (–)-xanthatin is shown. (**B**) MCF-7 cells (adherent) were treated with vehicle, (–)-xanthatin (**upper left** panel), etoposide (**upper right** panel), or salinomycin (0.025, 0.1, 1, 2.5, 10, and 25 µM) (**lower** panel) for 48 h, and cell viability was assessed. Data are presented as the mean ± S.E. (*n* = 6) percentage of the vehicle-treated control.

(–)-Xanthatin (Figure 1A), isolated from *Xanthium strumarium* L. (cocklebur), is a naturally occurring *exo*-methylene lactone group-containing compound. Cocklebur seeds can be squeezed to provide dietary oil in China, which is similar to safflower oil on the basis of its high content of linoleic acid [1,2]. In addition, *X. strumarium* is known to be a traditional herbal medicine (i.e., Cang Er Zi) in oriental countries. In recent years, experimental evidence has strongly suggested that (–)-xanthatin is an effective anti-proliferative molecule for a wide variety of cancer cells (in vitro) as well as an anti-tumorigenic molecule for in vivo cancer models [3,4,5,6,7,8,9,10,11,12]. Thus, (–)-xanthatin is attracting attention as a modality (a possible lead compound for cancer therapy) for the treatment of cancers. However, *X. strumarium* only produces trace amounts of the *exo*-methylene lactone compound. In order to further investigate and clarify the biological effects of (–)-xanthatin in cancer biology, it is important to obtain chemically synthesized pure (–)-xanthatin, not crude extracts. We originally developed a method for the total synthesis of (–)-xanthatin [13,14].

Growth arrest and DNA damage-inducible 45 (GADD45) family genes are known as critical stress sensors that mediate many cellular functions, including DNA repair, cell cycle control, and apoptosis [15]. Among the GADD45 family members, GADD45G is a functional tumor suppressor gene that is inactivated in different types of cancer cells, including breast cancer [16]. We reported that (–)-xanthatin abrogated the proliferation of highly aggressive breast cancer MDA-MB-231 cells via mechanisms involving (i) Topoisomerase IIα (Topo IIα) catalytic inhibition (i.e., accumulation of DNA damage) and (ii) the production of reactive oxygen species (ROS), which result in the re-activation of GADD45G expression; thus, (–)-xanthatin may be a selective inducer of GADD45G [3,4].

Cancer is a leading cause of death worldwide. Breast cancer, the most common cancer among women, is known to be a highly heterogeneous disease classified into four molecular subtypes (i.e., luminal A (the most common molecular subtype among patients), luminal B, human epidermal growth factor receptor 2-positive, and basal-like), with different prognoses and treatment responses [17]. Although significant development in cancer treatment has been seen during the past few decades, chemotherapy is still used to treat many types of cancer. In clinical settings, multidrug resistance (MDR) and the resulting ineffectiveness of chemotherapy drugs are responsible for over 90% of deaths in cancer patients [18]. Furthermore, the existence of cancer stem cells (CSCs) is considered another contributor to the failure of chemotherapy [19].

Although many effective anti-breast-cancer agents with different action mechanisms have been developed, breast cancer obtains phenotypes with the potential for relapse and resistance to chemotherapy [20,21,22]. Many researchers have investigated this issue, and recent studies have shown that cancer cells contain CSCs, a minor subpopulation of cells that (maybe) mandate the unwanted events described above [21,22]. Breast cancer stem cells (BCSCs) were initially identified in 2003 [23]. Salinomycin, traditionally used as an anti-coccidial drug, has been identified as a highly effective and selective abrogator of BCSCs via the high-throughput screening of ~16,000 compounds [20,21]. BCSCs have been enriched by the culturing of cells in non-adherent and non-differentiating conditions to form mammospheres [21]. Mammospheres from human breast cancer MCF-7 cells (luminal A subtype) may be more easily and reproducibly obtained than MDA-MB-231 cells (basal-like subtype) [24]. In addition, it currently remains unclear whether (–)-xanthatin activates the expression of GADD45G in MCF-7 cells, coupled with cell death. 

In the present study, we examined the effects of (–)-xanthatin and salinomycin (a positive control) on the formation of mammospheres derived from MCF-7 cells and found that (i) (–)-xanthatin reduced the viability of mammospheres more than salinomycin, (ii) (–)-xanthatin up-regulated the expression of *GADD45G* in a similar manner to salinomycin, and (iii) (–)-xanthatin down-regulated the expression of the stem cell markers (self-renewal regulatory factors) *Nanog*, *Oct4*, and *Sox2* [25]. We discuss the possible involvement of GADD45G, which may be negatively engaged in the viability of mammospheres, in strategies to abrogate these spheres.

## 2. Materials and Methods

### 2.1. Reagents

(–)-Xanthatin was completely synthesized based on a previously reported protocol [13,14] and was purified by HPLC; its purity (>95%) was confirmed by ^1^H- and ^13^C-NMR spectroscopy. Etoposide (purity > 98%) and salinomycin (purity > 97%) were purchased from FUJIFILM Wako Pure Chemical Corporation (Osaka, Japan) and Focus Biomolecules (Plymouth Meeting, PA, USA), respectively. (–)-Xanthatin was dissolved in ethanol, and etoposide and salinomycin were dissolved in dimethyl sulfoxide. 

### 2.2. Cell Cultures

The human breast cancer MCF-7 cells were obtained from the American Type Culture Collection (Rockville, MD, USA). Cell culture conditions (MCF-7 cells: adherent) and methods were based on previously described procedures [5,6,7,26]. In brief, MCF-7 cells were routinely cultured in phenol red-containing minimum essential medium α (MEMα) (FUJIFILM Wako Pure Chemical Corporation), supplemented with 10 mM 4-(2-hydroxyethyl)-1-piperazineethanesulfonic acid (HEPES), 5% fetal bovine serum (FBS), 100 U/mL of penicillin, and 100 µg/mL of streptomycin in a humidified incubator maintained at 37 °C and 5% CO_2_.

### 2.3. Cell Viability Analysis

To determine viability for MCF-7 cells (adherent), cell seeding and chemical treatment were performed as previously described [5]. After chemical treatment, CellTiter-Glo^®^ Luminescent Cell Viability Assay (Promega, Madison, WI, USA) was used to determine viability dependent on ATP availability, according to the manufacturer’s instructions.

### 2.4. Mammosphere Formation Assay and Chemical Treatment

MCF-7 cells were re-suspended in MammoCult™ Medium (+0.48 µg/mL hydrocortisone and 4 µg/mL heparin) (Stem Cell Technologies, Vancouver, BC, Canada), seeded onto an ultralow attachment culture plate (Corning, Acton, MA, USA) at 4 × 10^4^ cells/well, and incubated for 96 h. Mammospheres were treated with (–)-xanthatin, etoposide, and salinomycin for the times and at the final concentrations as indicated in each figure legend. Cell morphology analysis of mammospheres was performed as previously described [5]. Cell viability analysis of mammospheres was performed following the above-described method (See Section 2.3).

### 2.5. Preparation of Total RNA and Real-Time Reverse Transcription-Polymerase Chain Reaction (Real-Time RT-PCR) Analysis

Total RNA preparation and real-time RT-PCR were performed as previously described [5,26]. Primers for *GADD45G*, *Nanog*, *Oct4*, *Sox2*, and *β-actin* were determined in the previous studies [5,27]. The *GADD45G*, *Nanog*, *Oct4*, and *Sox2* mRNA expression levels were normalized to that of *β-actin*. 

### 2.6. Statistical Analysis

Differences were considered significant when *p* values were less than 0.05. The statistical significance of differences between the two groups was calculated using Student’s *t* test. Statistical significance for comparing more than two groups was determined using ANOVA with Dunnett’s post-hoc test. These calculations were performed using the StatView 5.0J software (SAS Institute Inc., Cary, NC, USA).

## 3. Results

**Figure 2:**
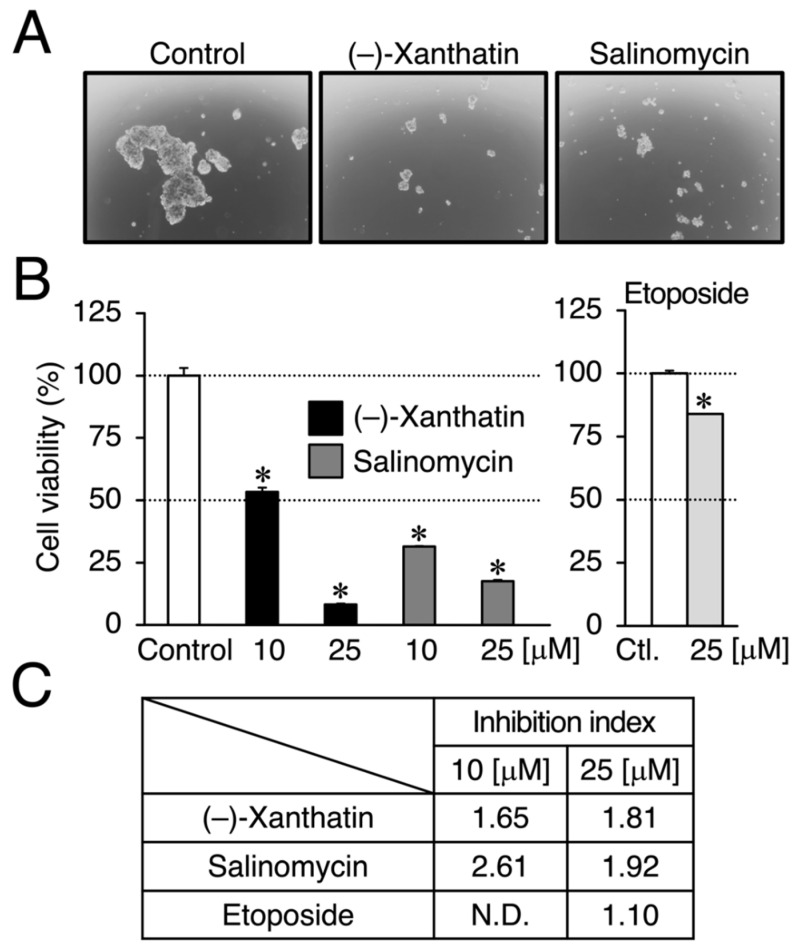
(–)-Xanthatin- and salinomycin-mediated suppression of the viability of mammospheres derived from MCF-7 cells. (**A**) Morphology of mammospheres treated with vehicle, 25 µM (–)-xanthatin, or 25 µM salinomycin for 72 h is shown (representative image). The images were taken at ×40 magnification. (**B**) Mammospheres were treated with vehicle, (–)-xanthatin (10 and 25 µM), salinomycin (10 and 25 µM) (**left** panels), or etoposide (25 µM) (**right** panel) for 72 h, and cell viability was assessed. Data are presented as the mean ± S.E. (*n* = 3) percentage of the vehicle-treated control. (**C**) The inhibition index, which is a ratio (%) of the viability between MCF-7 cells and mammospheres at the indicated concentrations of chemicals, is shown. When the inhibition index is greater than 1.0, the chemical exhibits the targeted abrogation of the viability of mammospheres. * Significant differences (*p* < 0.05) from the vehicle-treated control. N.D., not determined.

We compared the anti-proliferative effects exerted by (–)-xanthatin and salinomycin together with etoposide, an established anti-cancer agent that is a Topo IIα poison-type inhibitor as well as an ROS producer [6,7,28]. As shown in Figure 1B, (–)-xanthatin and salinomycin induced similar reductions in the viability of MCF-7 cells under adherent culture conditions (IC_50_ values: 16.1 and 18.4 µM, respectively) (upper left panel and lower panel). However, etoposide was shown to be entirely negative, even in the presence of 25 µM, the maximum concentration used (upper right panel). We then investigated the effects of (–)-xanthatin along with salinomycin and etoposide on the formation of MCF-7 mammospheres. The appearance of mammospheres was inhibited by (–)-xanthatin at 25 µM (Figure 2A). Quantification results indicated that (–)-xanthatin and salinomycin (10 and 25 µM) abolished the viability of MCF-7 mammospheres in a similar manner when the control incubation was set at 100% (Figure 2B, left panel). Furthermore, similar to the results shown in Figure 1C, etoposide was a less potent inhibitor for mammospheres than (–)-xanthatin and salinomycin, even though significant differences were observed (Figure 2B, right panel). In accordance with the results described above, the inhibition index, a ratio of cell viability (%), at the indicated concentrations of compounds between normal MCF-7 cells and mammospheres suggested that (–)-xanthatin and salinomycin, unlike etoposide, exhibited similar inhibition selectivity for mammospheres; however, the positive compound salinomycin was slightly more potent (Figure 2C).

**Figure 3:**
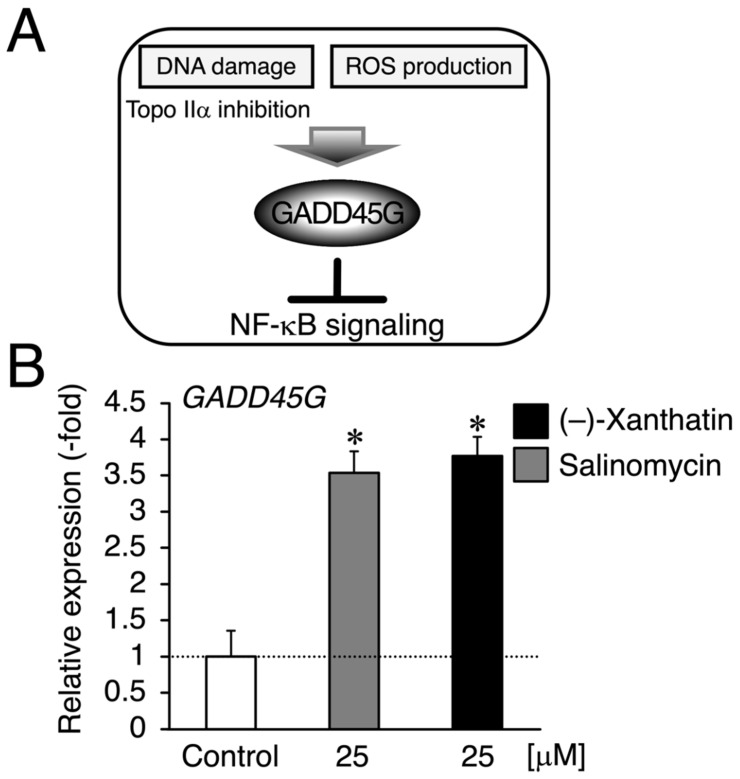
(–)-Xanthatin- and salinomycin-mediated up-regulation of the expression of *growth arrest and DNA damage-inducible 45 gamma* (*GADD45G*) in mammospheres derived from MCF-7 cells. (**A**) We reported that (–)-xanthatin has the ability to inhibit Topo IIα as a catalytic inhibitor as well as produce reactive oxygen species (ROS) associated with the induction of GADD45G in breast cancer cells [5,6,7,9,11]. (**B**) Real-time RT-PCR analysis of *GADD45G* in mammospheres after 72 h of the treatment with vehicle, 25 µM (–)-xanthatin, or 25 µM salinomycin. Data are presented as the mean ± S.E. (*n* = 3) of the fold induction compared to the vehicle-treated control. * Significant differences (*p* < 0.05) from the vehicle-treated control.

**Figure 4:**
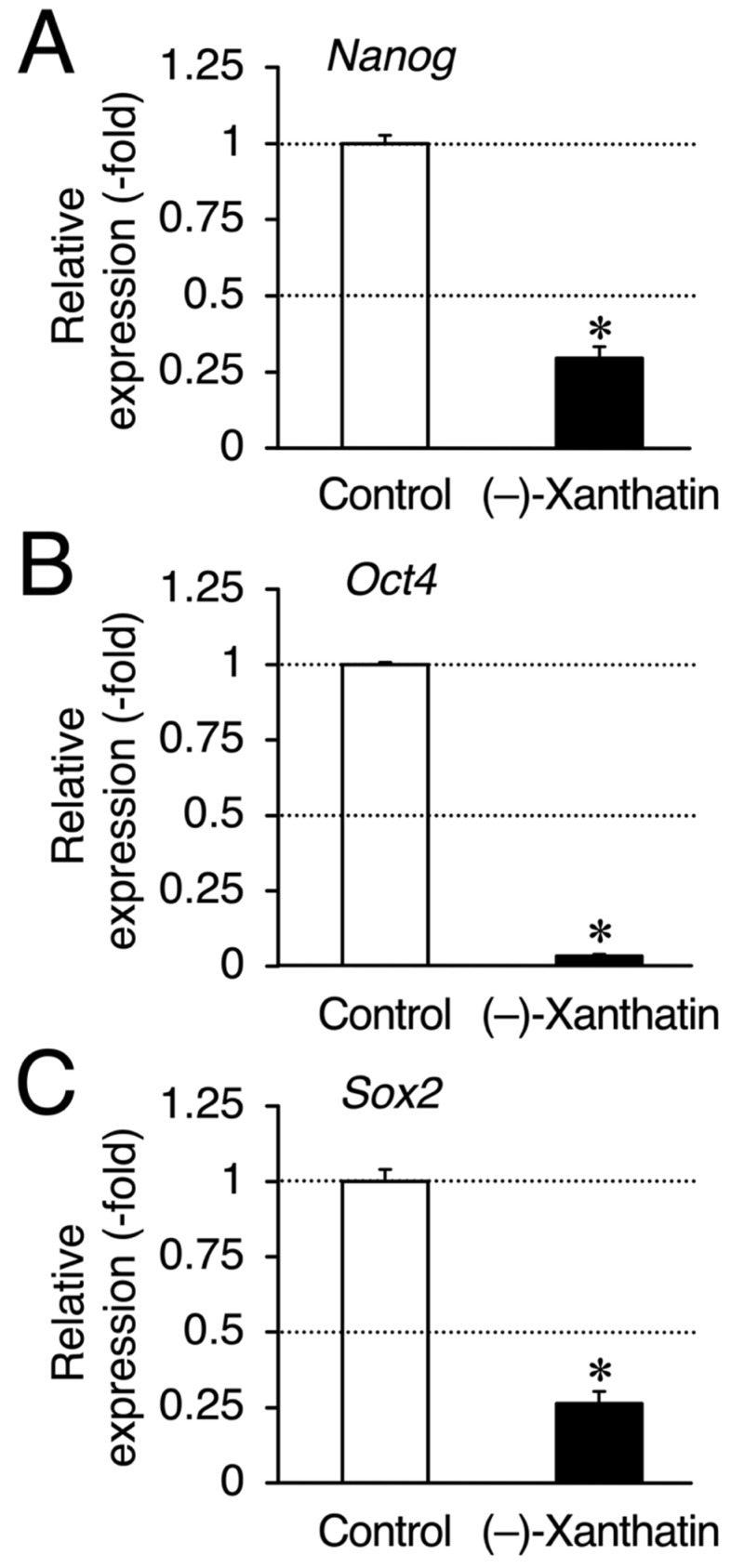
(–)-Xanthatin-mediated down-regulation of the expression of *Nanog*, *Oct4*, and *Sox2* in mammospheres derived from MCF-7 cells. Real-time RT-PCR analysis of *Nanog* (**A**), *Oct4* (**B**), and *Sox2* (**C**) in mammospheres after 72 h of the treatment with vehicle or 25 µM (–)-xanthatin. Data are presented as the mean ± S.E. (*n* = 3) of the fold induction compared to the vehicle-treated control. * Significant differences (*p* < 0.05) from the vehicle-treated control.

Since salinomycin satisfies the conditions of (i) DNA damage induction and (ii) ROS production, similar to (–)-xanthatin, a GADD45G inducer [5,6,7,9,15,29,30] (Figure 3A), we examined *GADD45G* gene expression in mammospheres treated with (–)-xanthatin and salinomycin. As expected, (–)-xanthatin and salinomycin significantly up-regulated the expression of *GADD45G* to the same extent (~3.5-fold) (Figure 3B), indicating that they share common pathway(s) to suppress the viability of mammospheres. An inverse relationship was observed between GADD45G and NF-κB for the control of cancer cell death; when GADD45G was activated, the other NF-κB-mediated signal was suppressed [31] (Figure 3A). Previous studies demonstrated that treatment with (–)-xanthatin inhibited the activity of NF-kB in human gastric carcinoma MKN45 cells [32]. NF-κB-mediated signaling is known to positively regulate the progression of breast cancer by suppressing death signals [31,33]; however, Karin’s research group also reported that NF-κB functions as a critical factor for the self-renewal of mammary tumor-initiating cells [34]. NF-κB has been shown to positively regulate three representative transcription factors aggressively involved in the self-renewal of BCSCs (mammospheres) (i.e., Nanog, Oct4, and Sox2) [25,35]. Since salinomycin down-regulates the expression of these three transcription factors in many lines of cancer stem cells, and based on the findings described above, (–)-xanthatin may reduce the viability of mammospheres via the GADD45G-mediated abrogation of NF-κB signaling, resulting in the down-regulation of Nanog, Oct4, and Sox2 in the spheres (Figure 3A). Thus, we analyzed the expression status of *Nanog*/*Oct4*/*Sox2* and found that all were maintained at very low levels following the exposure to (–)-xanthatin (<0.3-fold) (Figure 4).

## 4. Discussion

In the present study, etoposide was shown to be inactive as a killer of mammospheres (Figure 2B,C). Since etoposide inhibits Topo IIα or induces ROS production in cancer cells [6,7,28], a paradox exists for why the compound was not a killer of the spheres. When (–)-xanthatin and etoposide were compared from the standpoint of the Topo IIα inhibitory mode, the former was shown to be a catalytic inhibitor of Topo IIα, while the latter is a Topo IIα-poison type inhibitor [6,36]. Since the mechanisms used (i.e., Topo IIα catalytic inhibition vs. Topo IIα poison) to induce DNA damage by Topo IIα inhibition are a key factor evoking DNA damage responses coupled with GADD45G induction (Figure 3A), chemicals may need to exhibit the potential for Topo IIα catalytic inhibition, and thus, salinomycin may be categorized as a potential catalytic inhibitor of Topo IIα.

To date, (–)-parthenolide, which also has the moiety of active *exo*-methylene lactone (Figure 1A, (–)-xanthatin), is one of the molecules that has been established to selectively target human acute myelogenous leukemia stem cells; mechanistically, (–)-parthenolide is known to utilize NF-κB inhibition and ROS production [37]. If (–)-parthenolide induces *GADD45G*, this gene will be a candidate target for the selective abrogation of BCSCs. Although we did not obtain direct/rational evidence for the participation of GADD45G induced by (–)-xanthatin and salinomycin in the present study, this is the first study to demonstrate that (–)-xanthatin exhibits a similar BCSC (mammosphere)-targeting ability to salinomycin. Clearly, further studies are needed to establish that (–)-xanthatin in the *X. strumarium* is a killer of breast cancer.

## 5. Conclusions

Although it has been reported that (–)-xanthatin can exert anti-proliferative effects on breast cancer cells with unknown mechanisms, the findings of this study show that (–)-xanthatin targets mammospheres, which are enriched in breast cancer stem cells (BCSCs) derived from the luminal A type MCF-7 cell line (positive for estrogen and progesterone receptors). Importantly, (–)-xanthatin displayed the killing potential for mammospheres, comparable to salinomycin, an established killer of BCSCs in experimental settings. It has been reported by the American Cancer Society that among patients with estrogen receptor α-positive breast tumors, approximately 50% of recurrences occurred 5 years after initial diagnosis. In addition to 17β-estradiol-lowering agents, it is necessary to develop agents targeting BCSCs in clinical situations. These taken together, (–)-xanthatin might be a candidate molecule for suppression of breast cancer. Further research is warranted on (–)-xanthatin’s anti-proliferation effects in detail.

## Data Availability

Not applicable.

## References

[1] Kamboj A., Saluja A. (2010). Phytopharmacological review of *Xanthium strumarium* L. (Cocklebur). Int. J. Green Pharm..

[2] Fan W., Fan L., Peng C., Zhang Q., Wang L., Li L., Wang J., Zhang D., Peng W., Wu C. (2019). Traditional uses, botany, phytochemistry, pharmacology, pharmacokinetics and toxicology of *Xanthium strumarium* L.: A review. Molecules.

[3] Roussakis C., Chinou I., Vayas C., Harvala C., Verbist J.F. (1994). Cytotoxic activity of xanthatin and the crude extracts of *Xanthium strumarium*. Planta Med..

[4] Ramírez-Erosa I., Huang Y., Hickie R.A., Sutherland R.G., Barl B. (2007). Xanthatin and xanthinosin from the burs of *Xanthium strumarium* L. as potential anticancer agents. Can. J. Physiol. Pharmacol..

[5] Takeda S., Matsuo K., Yaji K., Okajima-Miyazaki S., Harada M., Miyoshi H., Okamoto Y., Amamoto T., Shindo M., Omiecinski C.J. (2011). (–)-Xanthatin selectively induces GADD45γ and stimulates caspase-independent cell death in human breast cancer MDA-MB-231 cells. Chem. Res. Toxicol..

[6] Takeda S., Noguchi M., Matsuo K., Yamaguchi Y., Kudo T., Nishimura H., Okamoto Y., Amamoto T., Shindo M., Omiecinski C.J. (2013). (–)-Xanthatin up-regulation of the GADD45γ tumor suppressor gene in MDA-MB-231 breast cancer cells: Role of topoisomerase IIα inhibition and reactive oxygen species. Toxicology.

[7] Takeda S., Nishimura H., Koyachi K., Matsumoto K., Yoshida K., Okamoto Y., Amamoto T., Shindo M., Aramaki H. (2013). (–)-Xanthatin induces the prolonged expression of c-Fos through an *N*-acetyl-L-cysteine (NAC)-sensitive mechanism in human breast cancer MDA-MB-231 cells. J. Toxicol. Sci..

[8] Li W.D., Wu Y., Zhang L., Yan L.G., Yin F.Z., Ruan J.S., Chen Z.P., Yang G.M., Yan C.P., Zhao D. (2013). Characterization of xanthatin: Anticancer properties and mechanisms of inhibited murine melanoma in vitro and in vivo. Phytomedicine.

[9] Takeda S., Okajima S., Miyoshi H., Koyachi K., Matsumoto K., Shindo M., Aramaki H. (2015). (–)-Xanthatin-mediated marked up-regulation of RhoB, a sensor for damaged DNA. Fundam. Toxicol. Sci..

[10] Yu Y., Yu J., Pei C.G., Li Y.Y., Tu P., Gao G.P., Shao Y. (2015). Xanthatin, a novel potent inhibitor of VEGFR2 signaling, inhibits angiogenesis and tumor growth in breast cancer cells. Int. J. Clin. Exp. Pathol..

[11] Takeda S., Okajima S., Noguchi M., Miyoshi H., Koyachi K., Matsumoto K., Shindo M., Aramaki H. (2016). Possible involvement of FosB in (–)-xanthatin-mediated anti-proliferative effects in human cancer MDA-MB-231 cells. Fundam. Toxicol. Sci..

[12] Tao L., Sheng X., Zhang L., Li W., Wei Z., Zhu P., Zhang F., Wang A., Woodgett J.R., Lu Y. (2016). Xanthatin anti-tumor cytotoxicity is mediated via glycogen synthase kinase-3β and β-catenin. Biochem. Pharmacol..

[13] Matsuo K., Ohtsuki K., Yoshikawa T., Shisho K., Yokotani-Tomita K., Shindo M. (2010). Total synthesis of xanthanolides. Tetrahedron.

[14] Matsumoto K., Koyachi K., Shindo M. (2013). Asymmetric total syntheses of xanthatin and 11,13-dihydroxanthatin using a stereocontrolled conjugate allylation to γ-butenolide. Tetrahedron.

[15] Humayun A., Fornace A.J. (2022). GADD45 in stress signaling, cell cycle control, and apoptosis. Adv. Exp. Med. Biol..

[16] Zhang X., Li Y., Ji J., Wang X., Zhang M., Li X., Zhang Y., Zhu Z., Ye S.-D., Wang X. (2021). Gadd45g initiates embryonic stem cell differentiation and inhibits breast cell carcinogenesis. Cell death Discov..

[17] Wesolowski R., Ramaswamy B. (2011). Gene expression profiling: Changing face of breast cancer classification and management. Gene Expr..

[18] Wang X., Zhang H., Chen X. (2019). Drug resistance and combating drug resistance in cancer. Cancer Drug Resist..

[19] Zhang Q., Feng Y., Kennedy D. (2017). Multidrug-resistant cancer cells and cancer stem cells hijack cellular systems to circumvent systemic therapies, can natural products reverse this?. Cell. Mol. Life Sci..

[20] Mani S.A., Guo W., Liao M.J., Eaton E.N., Ayyanan A., Zhou A.Y., Brooks M., Reinhard F., Zhang C.C., Shipitsin M. (2008). The epithelial-mesenchymal transition generates cells with properties of stem cells. Cell.

[21] Gupta P.B., Onder T.T., Jiang G., Tao K., Kuperwasser C., Weinberg R.A., Lander E.S. (2009). Identification of selective inhibitors of cancer stem cells by high-throughput screening. Cell.

[22] Ma C.X., Reinert T., Chmielewska I., Ellis M.J. (2015). Mechanisms of aromatase inhibitor resistance. Nat. Rev. Cancer.

[23] Al-Hajj M., Wicha M.S., Benito-Hernandez A., Morrison S.J., Clarke M.F. (2003). Prospective identification of tumorigenic breast cancer cells. Proc. Natl. Acad. Sci. USA.

[24] Manuel Iglesias J., Beloqui I., Garcia-Garcia F., Leis O., Vazquez-Martin A., Eguiara A., Cufi S., Pavon A., Menendez J.A., Dopazo J. (2013). Mammosphere formation in breast carcinoma cell lines depends upon expression of E-cadherin. PLoS ONE.

[25] Morrison B.J., Schmidt C.W., Lakhani S.R., Reynolds B.A., Lopez J.A. (2008). Breast cancer stem cells: Implications for therapy of breast cancer. Breast Cancer Res..

[26] Hirao-Suzuki M., Koga T., Sakai G., Kobayashi T., Ishii Y., Miyazawa H., Takiguchi M., Sugihara N., Toda A., Ohara M. (2020). Fatty acid 2-hydroxylase (FA2H) as a stimulatory molecule responsible for breast cancer cell migration. Biochem. Biophys. Res. Commun..

[27] Zhang X., Yalcin S., Lee D.F., Yeh T.Y., Lee S.M., Su J., Mungamuri S.K., Rimmelé P., Kennedy M., Sellers R. (2011). FOXO1 is an essential regulator of pluripotency in human embryonic stem cells. Nat. Cell. Biol..

[28] Kurosu T., Fukuda T., Miki T., Miura O. (2003). BCL6 overexpression prevents increase in reactive oxygen species and inhibits apoptosis induced by chemotherapeutic reagents in B-cell lymphoma cells. Oncogene.

[29] Kim J.H., Chae M., Kim W.K., Kim Y.J., Kang H.S., Kim H.S., Yoon S. (2011). Salinomycin sensitizes cancer cells to the effects of doxorubicin and etoposide treatment by increasing DNA damage and reducing p21 protein. Br. J. Pharmacol..

[30] Kim K.Y., Park K.I., Kim S.H., Yu S.N., Lee D., Kim Y.W., Noh K.T., Ma J.Y., Seo Y.K., Ahn S.C. (2017). Salinomycin induces reactive oxygen species and apoptosis in aggressive breast cancer cells as mediated with regulation of autophagy. Anticancer Res..

[31] Zerbini L.F., Wang Y., Czibere A., Correa R.G., Cho J.Y., Ijiri K., Wei W., Joseph M., Gu X., Grall F. (2004). NF-κB-mediated repression of growth arrest- and DNA-damage-inducible proteins 45α and γ is essential for cancer cell survival. Proc. Natl. Acad. Sci. USA.

[32] Zhang L., Tao L., Ruan J., Li W., Wu Y., Yan L., Zhang F., Fan F., Zheng S., Wang A. (2012). Xanthatin induces G2/M cell cycle arrest and apoptosis in human gastric carcinoma MKN-45 cells. Planta Med..

[33] Zerbini L.F., Libermann T.A. (2005). Life and death in cancer. GADD45 and are critical regulators of NF-κB mediated escape from programmed cell death. Cell Cycle.

[34] Cao Y., Luo J.L., Karin M. (2007). IκB kinase α kinase activity is required for self-renewal of ErbB2/Her2-transformed mammary tumor-initiating cells. Proc. Natl. Acad. Sci. USA.

[35] Liu M., Sakamaki T., Casimiro M.C., Willmarth N.E., Quong A.A., Ju X., Ojeifo J., Jiao X., Yeow W.S., Katiyar S. (2010). The canonical NF-κB pathway governs mammary tumorigenesis in transgenic mice and tumor stem cell expansion. Cancer Res..

[36] Takeda S., Yaji K., Matsumoto K., Amamoto T., Shindo M., Aramaki H. (2014). Xanthocidin derivatives as topoisomerase IIα enzymatic inhibitors. Biol. Pharm. Bull..

[37] Guzman M.L., Rossi R.M., Karnischky L., Li X., Peterson D.R., Howard D.S., Jordan C.T. (2005). The sesquiterpene lactone parthenolide induces apoptosis of human acute myelogenous leukemia stem and progenitor cells. Blood.

